# An Epithelial Serine Protease, AgESP, Is Required for *Plasmodium* Invasion in the Mosquito *Anopheles gambiae*

**DOI:** 10.1371/journal.pone.0035210

**Published:** 2012-04-11

**Authors:** Janneth Rodrigues, Giselle A. Oliveira, Michalis Kotsyfakis, Rajnikant Dixit, Alvaro Molina-Cruz, Ryan Jochim, Carolina Barillas-Mury

**Affiliations:** Laboratory of Malaria and Vector Research, National Institute of Allergy and Infectious Diseases, National Institutes of Health, Rockville, Maryland, United States of America; Centro de Pesquisas René Rachou, Brazil

## Abstract

**Background:**

*Plasmodium* parasites need to cross the midgut and salivary gland epithelia to complete their life cycle in the mosquito. However, our understanding of the molecular mechanism and the mosquito genes that participate in this process is still very limited.

**Methodology/Principal Findings:**

We identified an *Anopheles gambiae* epithelial serine protease (AgESP) that is constitutively expressed in the submicrovillar region of mosquito midgut epithelial cells and in the basal side of the salivary glands that is critical for *Plasmodium* parasites to cross these two epithelial barriers. AgESP silencing greatly reduces *Plasmodium berghei* and *Plasmodium falciparum* midgut invasion and prevents the transcriptional activation of gelsolin, a key regulator of actin remodeling and a reported *Plasmodium* agonist. AgESP expression is highly induced in midgut cells invaded by *Plasmodium*, suggesting that this protease also participates in the apoptotic response to invasion. In salivary gland epithelial cells, AgESP is localized on the basal side–the surface with which sporozoites interact. AgESP expression in the salivary gland is also induced in response to *P. berghei* and *P. falciparum* sporozoite invasion, and AgESP silencing significantly reduces the number of sporozoites that invade this organ.

**Conclusion:**

Our findings indicate that AgESP is required for *Plasmodium* parasites to effectively traverse the midgut and salivary gland epithelial barriers. *Plasmodium* parasites need to modify the actin cytoskeleton of mosquito epithelial cells to successfully complete their life cycle in the mosquito and AgESP appears to be a major player in the regulation of this process.

## Introduction

Malaria, an infectious disease caused by *Plasmodium* parasites, affects 247 million people every year. The *Anopheles gambiae* mosquito is the major vector of human malaria in sub-Saharan Africa, where most malaria episodes (86%) and deaths (91%) occur [1]. Mosquitoes become infected when they ingest blood from a vertebrate host that contains *Plasmodium* gametocytes. Zygotes are formed following fertilization in the midgut lumen and then mature into a motile form, the ookinete.

The mosquito midgut epithelium comprises a monolayer of columnar epithelial cells with an apical microvillar surface that faces the gut lumen and an intricate permeable membranous labyrinth on the basal side, which is bathed in hemolymph [2]. *Plasmodium* ookinetes interact with the luminal surface of the midgut and traverse epithelial cells without forming a vacuole [3], coming in direct contact with the cytoplasm of the invaded cell and causing irreversible damage that leads to apoptosis [4]–[6]. Ookinete midgut invasion causes differential regulation of more than 7% of the *An. gambiae* midgut transcriptome, including several genes that mediate reorganization of the actin cytoskeleton [7]. A functional screen of 11 candidate genes involved in cytoskeleton dynamics identified 4 genes that affect *Plasmodium* infection [7]. Silencing gelsolin or F-actin capping protein (CP) decreased *P. berghei* infection, while ciboulot or Wiskott-Aldrich syndrome protein (WASP) silencing had the opposite effect, enhancing infection [7]. These studies indicated that there are critical interactions between *Plasmodium* parasites and the cytoskeleton of midgut epithelial cells that determine the fate of ookinetes in the mosquito. When ookinetes emerge from epithelial cells, they come in contact with the basal lamina and transform into oocysts. During this stage, parasites form a capsule, multiply continuously, and eventually release hundreds of sporozoites into the circulating hemolymph. Sporozoites must cross a second barrier–the salivary gland (SG) epithelium–before they can reach the salivary duct. Unlike ookinetes, sporozoites invade the basal side of the SG epithelial cells by forming a transient parasitophorous vacuole [8]. Malaria transmission takes place when an infected mosquito takes a blood meal and injects mature sporozoites into the vertebrate host.

In mosquitoes, serine proteases participate in blood digestion [9]–[11] and have also been implicated in antiplasmodial immunity [12]–[14]. Serine proteases can also activate signal transduction pathways by proteolytic cleavage of specific target proteins [15]. A previous study identified a trypsin-like serine protease that is differentially expressed in response to *Plasmodium* infection between naturally occurring susceptible and refractory *Anopheles culicifacies* mosquitoes [13]. In this study, we characterized the putative *An. gambiae* ortholog of this protease. Our studies revealed that this epithelial serine protease (AgESP) has a unique subcellular localization, regulates expression of gelsolin (an actin-binding protein involved in remodeling of the cytoskeleton) in midgut epithelial cells, and is required for *Plasmodium* midgut and SG invasion.

## Results

### AgESP cDNA Sequence, Predicted Protein Sequence, and Tertiary Structure

**Figure 1:**
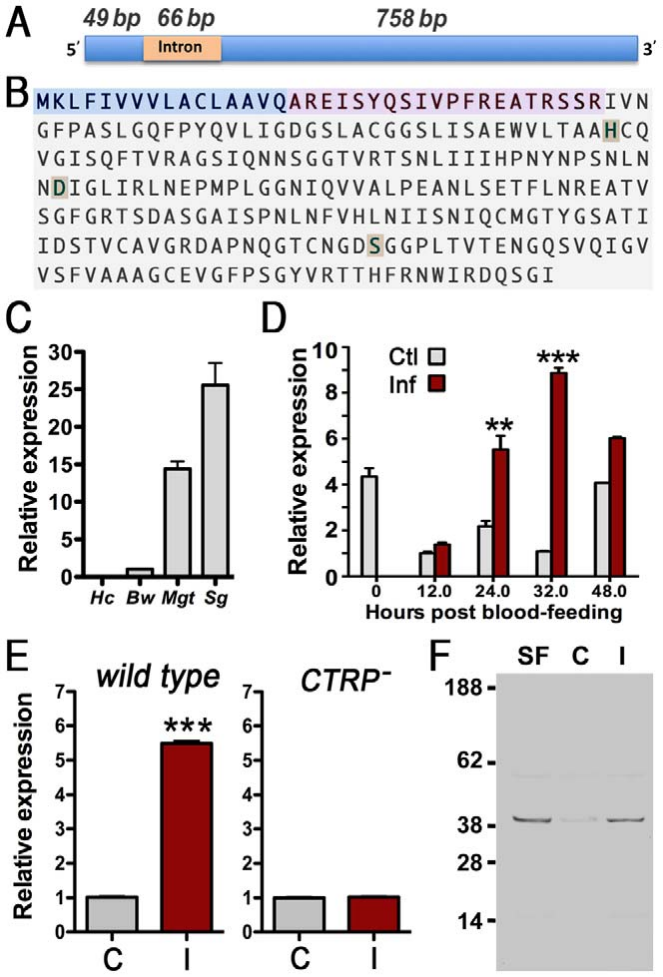
AgESP gene structure and expression. (A) Diagram of AgESP cDNA with exons shown in blue and the intron in orange. The size of each region is indicated in base pairs (bp). (B) Deduced amino acid sequence of AgESP protein showing the predicted signal peptide (light blue), the pro-peptide (pink), and the amino acids comprising the catalytic triad (H, D, and S; in beige). (C) Tissue-specific expression of AgESP mRNA in hemocytes (Hc), body wall (Bw), midgut (Mg), and salivary glands (Sg) of 5-day-old adult females. (D) AgESP mRNA levels in midguts of female mosquitoes fed on a healthy mouse (Ctl, control; grey bars) and *Plasmodium berghei*-infected (Inf; red bars) at different times post feeding (PF). (E) AgESP mRNA levels in midguts of mosquitoes fed on a healthy mouse (Ctl, control; grey bars) or infected with *P. berghei* (I, infected; red bars) ANKA 2.34 wild-type and *CTRP* ***^–^*** knockout parasites 24 h PF. The unpaired two-tailed *t*-test was used to compare the different experimental groups (*, *P* < 0.05; **, *P* < 0.01; ***, *P* < 0.001). (F) AgESP protein expression in midguts from sugar-fed (SF) females or 24 h after feeding on healthy (C, control) or *P. berghei*-infected (I) blood. All expression analysis was confirmed in 2–3 independent biological replicates.

The *An. gambiae* epithelial serine protease (*AgESP*) gene is located in the 3R chromosome of *An. gambiae* (AGAP010240-PA). The coding region of the AgESP cDNA was cloned and sequenced. The cDNA is 807-bp long and has a slightly different intron-exon boundary than the predicted sequence in the latest *An. gambiae* genome annotation, resulting in a transcript that is 21 bp shorter. The cDNA sequence (Accession No. GenBank HQ878386) revealed a transcript composed of two exons (49 bp and 758 bp) separated by a 66-bp intron (Fig. 1A). The predicted amino acid sequence codes for polypeptide of 268 amino acids (aa), including a 17-aa putative signal peptide (MKLFIVVVLACLAAVQA) (Fig. 1B, shaded in light blue) and a 19-aa pro-peptide (REISYQSIVPFREATRSSR) (Fig. 1B, shaded in pink).

### AgESP Expression is Induced by Plasmodium Midgut Invasion

The potential participation of AgESP in midgut epithelium responses to *Plasmodium* infection was investigated. AgESP mRNA is expressed at high levels in the midgut and salivary glands (SGs) of adult sugar-fed *An. gambiae* females (Fig. 1C). It is expressed at low levels in the body wall (with the fat body attached to it), but cannot be detected in circulating hemocytes (Fig. 1C). AgESP midgut mRNA level decrease by about 80% 12 h post feeding (PF), but increase in *P. berghei*-infected midguts at 24 and 32 h PF (Fig. 1D). This response was not observed when mosquitoes were fed on a mouse infected with the *P. berghei* CTRP knockout (KO) line that produces ookinetes unable to invade the midgut (Fig. 1E), indicating that ookinete invasion is required to induce AgESP midgut expression.

Recombinant AgESP was expressed in the *Escherichia coli* system, purified, and used to generate anti-AgESP polyclonal antibodies. AgESP protein is expressed in sugar-fed midguts as a single 40-kDa band (Fig. 1F) and follows an expression pattern similar to that of AgESP mRNA. AgESP protein expression also decreases to very low levels by 24 h PF in the midgut of mosquitoes fed on an uninfected mouse (Fig. 1F; C  =  control) and is induced in response to *Plasmodium* infection (Fig. 1F; I  =  infected).

**Figure 2:**
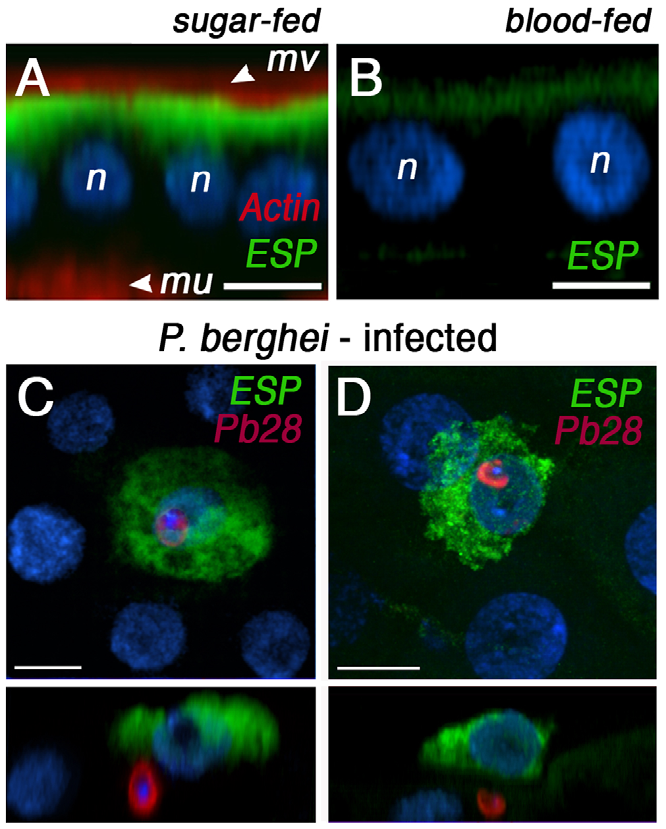
AgESP immunolocalization and expression in midguts infected with *Plasmodium berghei*. AgESP protein expression in midguts from (A) sugar-fed females or (B) 24 h post-feeding (PF) on healthy mouse. Nucleus (n), microvilli (mv), Basal lamina (mu). AgESP protein (green), actin (red), and nucleus (blue). (C, D) Immunofluorescence staining of *P. berghei*-infected midguts 28 h PF. AgESP protein (green), Pbs28 on the ookinete surface (red), and nucleus (blue). Top views are shown in the upper panels and the corresponding side views in the lower panels.

Immunofluorescence staining and confocal imaging revealed that AgESP is highly expressed in sugar-fed midguts (Fig. 2A). The protein is localized in the luminal side of the cell (Fig. 2A, shown in green) immediately underneath the microvilli (Fig. 2A). AgESP has a similar distribution in midgut cells 24 h PF on a healthy uninfected mouse, but the expression level is much lower than in sugar-fed females (Fig. 2B). AgESP protein expression is highly induced in epithelial cells invaded by *Plasmodium* ookinetes that have been damaged and are in the process of budding-off from the midgut (Fig. 2, C and D). In cells undergoing apoptosis, AgESP expression is no longer limited to the luminal side of the cell but is expressed throughout the cell cytoplasm (Fig. 2, C and D).

### AgESP is Required for Ookinete Invasion and Regulates Gelsolin Expression in the Midgut

**Figure 3:**
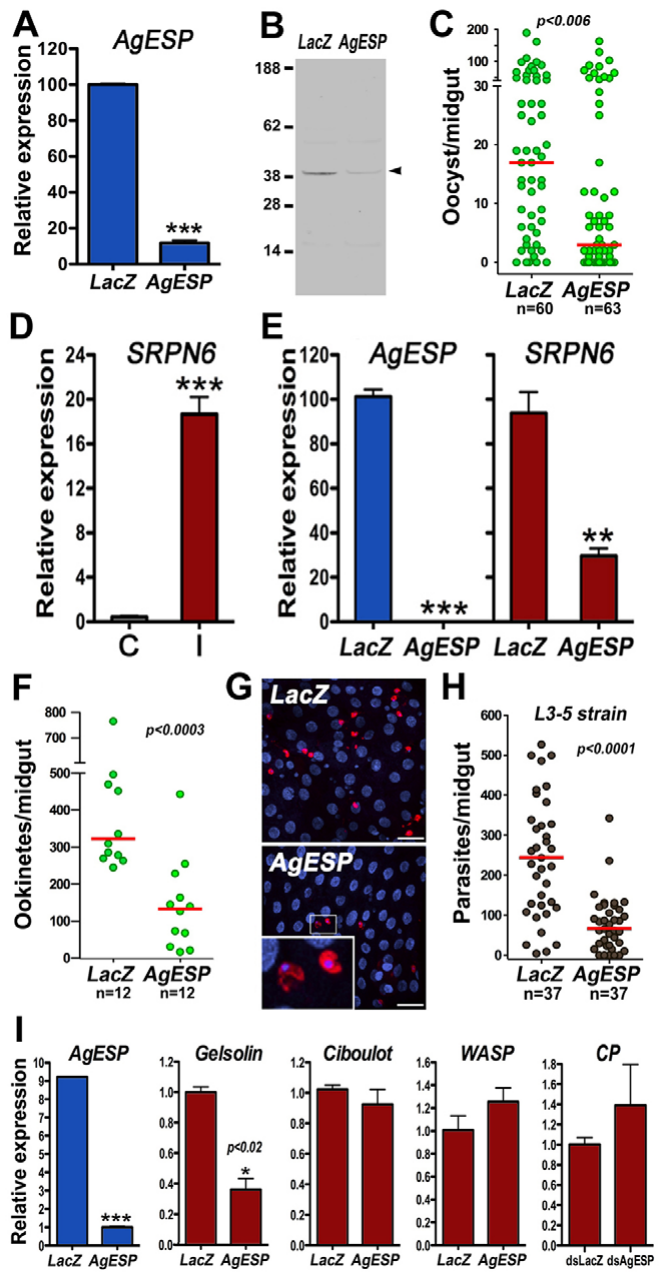
Effect of AgESP silencing on *Plasmodium berghei* infection. AgESP mRNA (A) and protein (B) silencing in the mosquito midgut by systemic dsRNA injection. (C) Effect of AgESP silencing on the number of *P. berghei* oocysts present in the midgut 7 days post infection. (D) Effect of *P. berghei* infection on SRPN6 expression. SRPN6 mRNA levels in midguts of mosquitoes fed on a healthy mouse (C, control; grey bar) or a *P. berghei-*infected mouse (I, infected; red bar). (E) Effect of AgESP silencing on expression of AgESP (blue bars) or SRPN6 (red bars) 24 h after ingestion of a *Plasmodium*-infected blood meal. (F) A significant reduction in number of parasites that invaded mosquito midgut was already observed in AgESP-silenced mosquitoes 28 h post feeding (PF). (G) Immunostaining of the ookinete surface with anti-Pb28 antibodies (red) and nuclear staining with DAPI (blue). There are fewer ookinetes in AgESP-silenced midguts, but no evidence of parasite fragmentation was observed. (H) Effect of AgESP silencing in the refractory *Anopheles gambiae* L35 strain on the number of melanized parasites (black dots). Each dot represents the number of parasites on an individual midgut. Live parasites are shown in green and melanized parasites in black. The red line indicates the median. Distributions were compared using the Mann-Whitney test. (I) Effect of silencing AgESP (blue bars) on the expression of gelsolin, Ciboulot, Wiskott-Aldrich syndrome protein (WASP), and F-actin capping protein (CP) 24 h PF on a *P. berghei*-infected mouse. The unpaired two-tailed *t*-test was used to compare the different experimental groups (*, *P* < 0.05; **, *P* < 0.01; ***, *P* < 0.001). All expression analysis was confirmed in 2–3 independent biological replicates. Infection phenotypes were confirmed in 2–3 independent experiments.

AgESP mRNA and protein expression was efficiently silenced by dsRNA injection (Fig. 3, A and B). AgESP silencing decreased the median number of oocysts present 7 days PF by 6-fold (*P* < 0.006) and the prevalence of infection from 85 to 65% (χ^2^; *P* < 0.001) (Fig. 3C).

As previously reported, expression of the serine protease inhibitor serpin 6 (SRPN6) is very low in uninfected midguts but is highly induced in response to ookinete invasion (Fig. 3D), making SRPN6 a sensitive molecular marker of cell invasion [16]. AgESP silencing reduced SRPN6 expression in *Plasmodium*-infected midguts 24 h PF by 70% (Fig. 3E), suggesting a reduction in ookinete midgut invasion.

To confirm that AgESP is required for ookinete midgut invasion, we determined the effect of AgESP silencing on the number of ookinetes present 28 h PF. The decrease in infection was already apparent (*P* < 0.0003) at this earlier time point, soon after most ookinetes have emerged from the midgut (Fig. 3F). Confocal microscopy revealed that although fewer ookinetes invaded the midgut of AgESP-silenced females, the parasites were intact and there was no evidence of ookinete fragmentation (Fig. 3G). AgESP was also silenced in the *An. gambiae* refractory (R) L35 strain, which melanizes parasites in the ookinete-to-oocyst transition as they emerge from the midgut and come in contact with the hemolymph [17]. AgESP silencing also reduced the numbers of parasites that invade the midgut in refractory females (Fig. 3H; *P* < 0.0001). All parasites were melanized in both the dsLacZ and dsAgESP-injected groups, indicating that AgESP does not participate in melanotic encapsulation. Together, these findings indicate that AgESP is required for *P. berghei* ookinetes to invade the midgut.

Ookinetes are known to interact directly with the cytoplasm of epithelial cells, because they do not form a parasitophorous vacuole as they traverse the midgut [3], [18]. We investigated whether AgESP could mediate interactions between *Plasmodium* ookinetes and the cytoskeleton of midgut epithelium cells. The effect of silencing AgESP on the expression of four genes–gelsolin, CP, ciboulot, and WASP–known to affect *P. berghei* infection in *An. gambiae* [7] was evaluated. AgESP silencing reduced gelsolin mRNA levels by up to 95% (47–95% in three independent experiments) in *Plasmodium*-infected midguts 24 h PF (Fig. 3I) but did not affect CP, ciboulot, or WASP expression. Gelsolin is an actin-binding protein that can cleave and cap the barbed ends of actin filaments [19]. Gelsolin [7] and AgESP (Fig. 3, C and F–H) silencing both reduce *P. berghei* infection, suggesting that AgESP may modulate cytoskeleton dynamics by regulating gelsolin expression.

**Figure 4:**
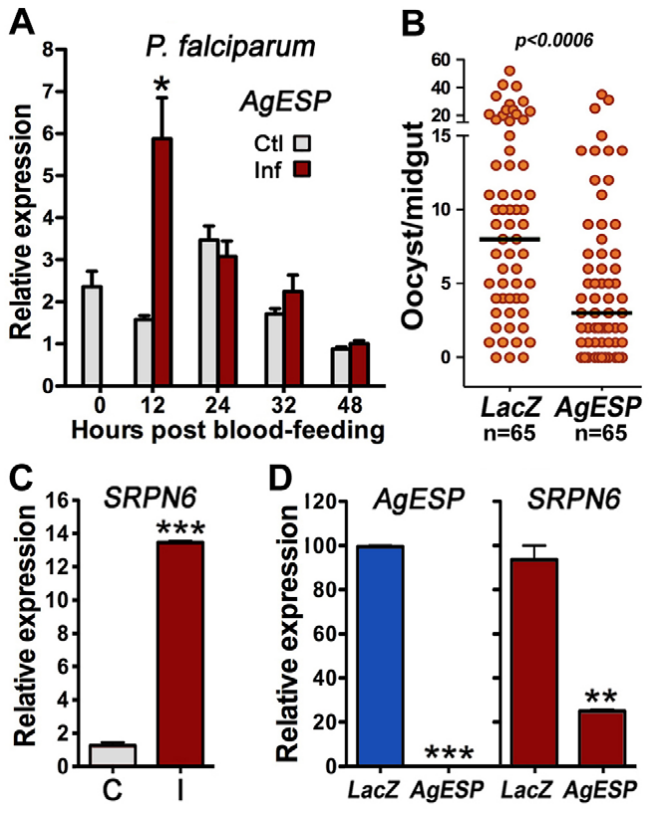
Effect of AgESP silencing on *Plasmodium falciparum* midgut infections. (A) AgESP mRNA levels in midguts from uninfected (Ctl, control; grey bars) and *P. falciparum* (3D7)-infected (Inf, infected; red bars) mosquitoes at different times post feeding (PF). (B) Effect of AgESP silencing on the number of *P. falciparum* (3D7) oocysts present 7 days PF. Each orange dot represents the number of live oocysts in an individual midgut; the black (**–**) line indicates the median. Distributions were compared using the Mann-Whitney test. (C) Effect of *P. falciparum* infection on SRPN6 expression. SRPN6 mRNA levels 12 h PF in midguts of mosquitoes fed uninfected blood (C, control; grey bars) or a *P. falciparum* gametocyte culture (I, infected; red bars). (D) Effect of AgESP silencing on midgut expression of AgESP (blue bars) or SRPN6 (red bars) in *P. falciparum*-infected female mosquitoes 18 h PF. All expression analysis was confirmed in 2–3 independent biological replicates. Infection phenotypes were confirmed in 2–3 independent experiments.

AgESP expression was also induced in the midguts of female mosquitoes infected with *P. falciparum* 12 h PF (Fig. 4A), and AgESP silencing reduced both the intensity (Fig. 4B; *P* < 0.0006) and the prevalence of infection with this human malaria parasite from 97% to 83% (χ^2^; *P* < 0.001) (Fig. 4B). SRPN6 expression is also induced in response to *P. falciparum* infection (Fig. 4C), and AgESP silencing (Fig. 4D, left panel) greatly reduces SRPN6 expression (Fig. 4D, right panel) (*P* < 0.01), indicating that AgESP also mediates *P. falciparum* midgut invasion.

### AgESP is Required for SG Invasion by Sporozoites

**Figure 5:**
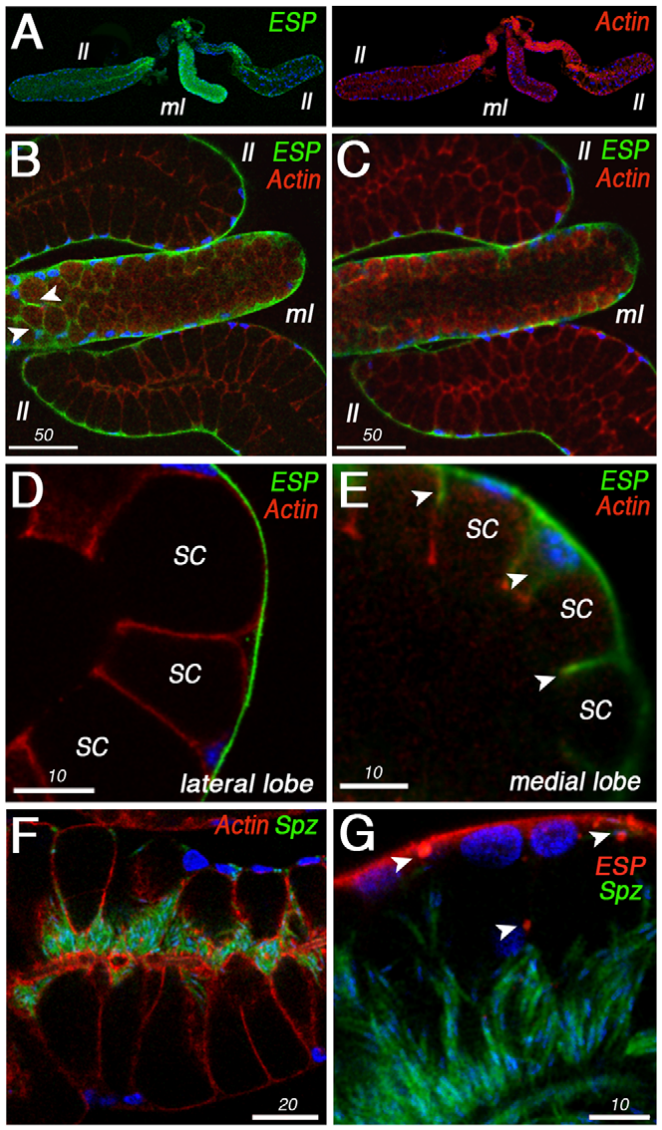
AgESP immunolocalization in *Anopheles gambiae* salivary glands (SGs). (A) AgESP is expressed in the distal region of the lateral lobes (ll) and medial lobes (ml), with a stronger staining in the medial lobe. AgESP protein (green) in the left panel and actin (red) in the right panel. (B, C) AgESP protein is expressed on the basal side of the distal lobes of the SG lobes. (D) In the lateral lobes, AgESP is only present on the basal surface of the epithelial cells lining the secretory cavities (SC). (E) In the medial lobes, AgESP is also present in the cytoplasm of epithelial cells as indicated by the white arrowheads in (B) and (E). (F) Actin staining in red and sporozoites, GFP in green, in *P. berghei*-infected SGs 18 days post feeding (PF). (G) Localization of ESP in red and sporozoites, GFP in green, in *P. berghei*-infected SGs (18 days PF). White arrowheads indicate the presence of ESP aggregates in infected SGs.

Ookinetes invade the midgut through the luminal side of the cell and presumably interact with AgESP protein present in the submicrovillar region during the invasion process. In contrast, sporozoites invade the SG epithelium through the basal side of the cell. The subcellular localization of AgESP in the SG was determined by immunofluorescence microscopy. In the SGs of adult females, AgESP is expressed in the distal region of the lateral lobes and medial lobes–the regions of the gland that are invaded by *Plasmodium* sporozoites–but cannot be detected in the proximal lobes (Fig. 5A). The staining is stronger in the medial lobes than in the lateral lobes (Fig. 5A). AgESP is expressed on the basal side of the SG (Fig. 5, B and C). Interestingly, this is the surface of the SG that sporozoites interact with during invasion. Single confocal sections indicate that, in the distal lateral lobes, AgESP is expressed on the basal side of the epithelial cells lining the secretory cavities (Fig. 5D). In the distal medial lobes, AgESP staining is strong on the basal side, but the protein is also present in the cell cytoplasm of the cells that surround the secretory cavities (Fig. 5, B and E, cytoplasmic staining is indicated by the arrowheads). No significant changes in the actin cytoskeleton were observed in SGs infected with sporozoites at 18 days post infection (Fig. 5F), and AgESP had a similar localization in infected as in uninfected glands (Fig. 5G). The only difference was the presence of AgESP aggregates in the infected glands (Fig. 5G, arrowheads), which are not observed in uninfected controls (Fig. 5, D and E). The AgESP aggregates in the infected salivary glands probably represent remnants of the invasion vesicles, suggesting that AgESP is internalized when sporozoites invade the cells.

**Figure 6:**
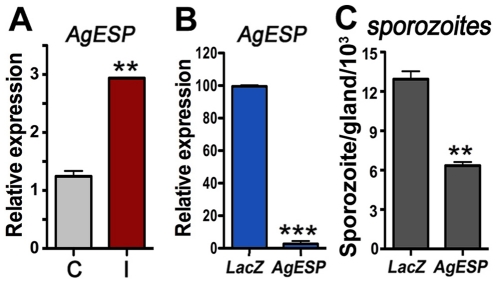
AgESP expression and effect of silencing on Plasmodium berghei salivary gland (SG) infection. (A) AgESP mRNA levels in SGs of mosquitoes fed on a healthy mouse (C, control; grey bars) or a *P. berghei-*infected mouse (I, infected; red bars) 18 days post feeding (PF). (B) Effect of AgESP dsRNA injection on AgESP expression (blue bars). Infected mosquitoes were injected with dsRNA 14 days PF, and mRNA levels were determined 4 days later. (C) Effect of AgESP silencing on the number of *P. berghei* sporozoites present in the SGs (black bars) 21 days PF. The unpaired two-tailed *t*-test was used to compare the different experimental groups (**, *P* < 0.01; ***, *P* < 0.001). All expression analysis was confirmed in 2–3 independent biological replicates. Infection phenotypes were confirmed in 2–3 independent experiments.

The potential role of AgESP in sporozoite SG invasion was investigated. SG infection with *P. berghei* (Fig. 6A) or *P. falciparum* (Fig. S1) sporozoites induced expression of AgESP mRNA 18 days PF. AgESP SG expression was efficiently silenced by injecting mosquitoes with dsRNA 14 days after they were infected with *P. berghei*, reducing endogenous mRNA levels 4 days after dsRNA injection by 95% (Fig. 6B). AgESP silencing significantly reduced the number *P. berghei* sporozoites present in the SG 21 days PF (*P* < 0.01) (Fig. 6C) but did not affect gelsolin expression (Fig. S2).

## Discussion

AgESP is constitutively expressed in the mosquito midgut and is localized in the submicrovillar region of epithelial cells, an area that ookinetes interact with during invasion. This protease is critical for *Plasmodium* midgut invasion, as silencing AgESP greatly reduces *P. berghei* and *P. falciparum* infection in *An. gambiae*. Transcriptional analysis of the responses of *An. gambiae* midgut to *P. berghei* infection [7] revealed that the most noticeable class of genes induced in response to ookinete invasion were those that modulate the architecture of the actin cytoskeleton. Silencing either gelsolin [7] or AgESP (Fig. 3C) results in a similar phenotype in which the intensity and prevalence of *Plasmodium* infection is greatly reduced. These observations suggest that the critical role of AgESP during ookinete invasion may be through induction of gelsolin expression, as AgESP silencing greatly reduces gelsolin expression (Fig. 3I) without affecting the expression of other genes thought to be involved in midgut cytoskeletal modifications in response parasite invasion, such as CP, Ciboulot or WASP.

The kinetics of AgESP induction in response to *Plasmodium* infection is different between *P. berghei* and *P. falciparum*-infected midguts. In *P. berghei* AgESP expression is induced at 24 h PF. This transcriptional response requires midgut invasion and SRPN6 expression, a sensitive marker of cell invasion, is also induced at this time. However, the induction of AgESP and SRPN6 expression is observed much earlier (12 h PF) in *P. falciparum*-infected midguts, suggesting that at least a few *P. falciparum* ookinetes may begin to invade the midgut earlier than previously thought. It is also possible that there is a long pre-invasion interaction of *P. falciparum* parasites with the midgut surface that triggers expression of these markers.

Apoptotic responses also trigger major rearrangements in the actin cytoskeleton [19]. For example, in vertebrates, gelsolin is cleaved by caspase-3, and microinjection of the cleaved gelsolin fragment that contains the actin-severing activity triggers rapid depolymerization of the actin cytoskeleton [20]. AgESP protein expression is low 24 h PF in the midgut of mosquitoes fed on a healthy mouse (Fig. 2, B and D), and expression is highly induced in response to *Plasmodium* infection (Fig. 2, B–F). This induction requires cell invasion, as it is not triggered by CTRP**^–^** *P. berghei* parasites, which form ookinetes that do no invade the midgut (Fig. 2C). Furthermore, induction of AgESP expression in the midgut of *P. berghei*-infected females peaks at 32 h PF (Fig. 2C), a time when many invaded cells are undergoing apoptosis and AgESP protein is highly expressed in the cytoplasm of cells undergoing apoptosis (Fig. 2, E and F). This suggests that, besides its involvement in ookinete midgut invasion, AgESP probably has a second function and may also mediate the apoptotic response triggered by parasite invasion. Interestingly, the induction of AgESP expression is more transient in *P. falciparum*-infected midguts, as it is no longer observed between 24 and 48 h PF, a time when invaded midgut cells are known to be undergoing apoptosis [21]. This suggests that there may also be functional differences in the apoptotic responses of midgut cells to invasion by theses two parasite species.

SRPN6 is a well-studied marker of midgut [16] and salivary gland [22] parasite invasion. Silencing SRPN6 does not affect AgESP expression (data not shown), indicating that AgESP acts upstream of SRPN6. The fact that silencing AgESP significantly reduces SRPN6 expression in the midgut as well as the salivary gland, indicates that AgESP is required for SRPN6 to be induced. Although it is possible that SRPN6 could act as a direct inhibitor of AgESP, we believe this unlikely because silencing SRPN6 does not enhance *P. berghei* infection in *A. gambiae* G3 females [22]. The effect of AgESP on SRPN6 expression could be indirect, as SRPN6 is not expressed constitutively in the midgut or salivary gland, and the lack of invasion when AgESP is absent may prevent activation of the signals that mediates the induction of SRPN6 by the parasite.

Immunofluorescence staining revealed that AgESP protein is abundant on the luminal side of the mosquito midgut (Fig. 2, A and B), while in the SGs, AgESP is present on the basal surface of the distal lobes (Fig.5, B–E). AgESP is constitutively expressed in high abundance in the distal lobes, unlike SRPN6 [22], which is detected only at sites of sporozoite invasion in the lateral lobes. Sporozoite SG invasion is asynchronous, taking place between 13 and 19 days post infection, and is facilitated by specific receptor-ligand interactions [23]. AgESP silencing in SGs decreased the number of sporozoites, indicating that this protease is also involved in sporozoite SG invasion. Unlike midgut cells, each SG cell is invaded by numerous sporozoites through the formation of parasitophorous vacuoles [3]. An increase in the number of microtubules in the basal region of infected cells has been documented in transmission electron microscopy studies, but no signs of apoptosis have been observed [24], probably due to the “gentler” mechanism of parasite invasion in the SG. The fact that AgESP silencing does not affect gelsolin expression in the SG suggests that this protease promotes sporozoite invasion through a mechanism different from that in the midgut, probably not involving changes in the cytoskeletal architecture mediated by the induction of gelsolin expression.

Together, our findings indicate that AgESP is expressed on the surface of the two epithelial barriers that *Plasmodium* parasites need to cross to complete their life cycle in the mosquito. *Plasmodium* parasites activate a cascade of events in mosquito epithelial cells to traverse these organs, and AgESP is a major player in the regulation of the invasion process. The recent development of a synthetic homing endonuclease gene drive system in *An. gambiae* [25] opens the possibility of spreading the disruption of genes such as AgESP in natural populations. This strategy could result in mosquitoes with reduced ability to transmit malaria because *Plasmodium* parasites would no longer be able to invade the midgut or the salivary glands efficiently.

## Materials and Methods

### Ethics Statement

Public Health Service Animal Welfare Assurance #A4149-01 guidelines were followed according to the National Institutes of Health (NIH) Office of Animal Care and Use (OACU). These studies were done according to the NIH animal study protocol (ASP) approved by the NIH Animal Care and User Committee (ACUC), with approval ID ASP-LMVR5.

### Mosquito Rearing

*An. gambiae* (G3 strain) mosquitoes were raised at 27°C, 80% humidity under a 12-h light/dark cycle and maintained on a 10% Karo syrup solution during adult stages.

### Synthesis of Double-stranded (ds) RNA

A 500-bp cDNA fragment from the AgESP cDNA was amplified using the primers Fwd: 5′-CTA ACCGCTGCTCACTGTCA-3′ and Rev: 5′-ACGAAGGACACCACACCAAT-3′ and cloned into the pCR®II-TOPO® vector (Invitrogen, Carlsbad, CA, USA). T7 promoters were introduced at both ends of this fragment using vector primers M13 Fwd-5′-CTCGAGTAATACGACTCACTATAGGGC AGGAAACAGCTATGAC-3′ and M13 Rev: 5′-CTCGAGTAATACGACTCACTATAGGGGCCAGTGTGA TGGATATCTGC-3′. The PCR product was used as template for dsRNA synthesis using the MEGAscript RNAi kit following the instructions of the manufacturer (Ambion, Austin, TX, USA). The eluted dsRNA was further purified and concentrated to 3 µg/µl in sterile H_2_O using a Microcon YM-100 filter (Millipore, Bedford, MA, USA). A similar strategy was used to synthesize dsLacZ using primers: LacZ Fwd: 5′-GAGTCA GTGAGCGAGGAAGC-3′ and LacZ Rev: 5′-TATCCG CTCACAATTCCACA-3′. The pIISRPN6.3 plasmid containing two T7 promoters flanking the cloned SRPN6 fragment, generously provided by Dr. Kristin Michel, was used as a template to generate a PCR product using primers with T7 promoter regions; Fwd: 5′-TAATACGACTCACTATAGGGG GCAACGCTCACCGGCAAGATG-3′ and Rev: 5′-TAATACGACTCACTATAGGGGGAGCGGCGCACTAA ATAAATAACGAG-3′. This product was used to synthesize SRPN6 dsRNA using the same protocol described above.

### Gene Silencing in An. Gambiae

To silence AgESP and SRPN6 midgut expression, 4- to 5-day-old female mosquitoes were cold anesthetized, and 69 nl of 3 µg/µl of dsRNA were injected into the thorax using a nano-injector (Nanoject; Drummond Scientific, Broomall, PA, USA). The control group was injected with dsLacZ. Mosquitoes were allowed to recover for 36 h post injection (PI) and then fed on a mouse infected with *P. berghei* or on a *P. falciparum* gametocyte culture provided in an artificial membrane feeding system. Mosquitoes were dissected 24 h PI to determine the effect of midgut AgESP silencing on expression of various genes or at 8 days PI to determine the infection level by counting oocysts. Silencing validation was done using real-time PCR relative to controls injected with dsLacZ as described below. To silence AgESP in the SGs, infected mosquitoes (14 day PI) were separated into two groups, cold anesthetized, and injected systemically with dsLacZ and dsAgESP by intrathoracic injection. Silencing efficiency was determined 2 to 3 days PI in pools of 10–14 SGs per treatment.

### Recombinant AgESP Protein Expression and Antibody Production

A 750-bp product of the coding region of AgESP was amplified from midgut cDNA using the primers Fwd 5-′ATCGTTCCATTTCGTGAAGC-3′ and Rev 5′-TCATTAGAAATGGGTGGTGCGTA CAT-3′, cloned into the pCR®T7/NT-TOPO® expression vector (Invitrogen) that includes an N-terminal His-tag, and transformed into the *E. coli* strain BL21(DE3) pLysS *E. coli* (Invitrogen). AgESP protein expression was induced by adding 1 mM IPTG to the bacterial culture and harvesting the cells 4 h later. The recombinant AgESP protein was insoluble and precipitated with the bacterial inclusion bodies. The protein was purified under denaturing conditions by affinity chromatography on a Ni-NTA resin following the manufacturer’s instructions (Qiagen, Valencia, CA, USA) and was injected into rabbits to generate polyclonal antibodies at Primm Biotech, Inc. (Cambridge, MA, USA) using a standardized immunization protocol followed by the company.

### Western Blot Analysis

Midguts were dissected from sugar-fed females or from mosquitoes fed on blood from a healthy or an infected mouse. The blood meal was removed and the tissue homogenized in PBS buffer containing protease inhibitors (complete protein inhibitor cocktail tablets; Roche, Mannheim, Germany). Equal amounts of protein (10 µg) from the different samples were loaded onto a NuPAGE® Novex Bis-Tris Gel (Invitrogen) and subjected to SDS electrophoresis under reducing conditions. Proteins were transferred to a nitrocellulose membrane (Invitrogen), which was incubated with freshly prepared 1 mM levamisole (Sigma, St. Louis, MO, USA) solution (in water) for 1 h to inhibit any internal phosphatase activity. Membrane was washed and then blocked with 5% BSA plus TBS-Tween (10 mM Tris-HCl, 150 mM NaCl, 0.05% Tween 20, pH7.6) at 4°C overnight. After three washes with TBS-Tween of 10 min each, the membrane was incubated with anti-AgESP rabbit antibody (1∶300) in 3% BSA plus TBS-Tween at 4°C for 3–4 h. Membranes were washed and incubated for 2 h at room temperature with secondary anti-rabbit alkaline phosphatase-conjugated antibody (1∶7500) (CalBiochem, San Diego, CA, USA). AgESP was detected using Western Blue substrate for alkaline phosphatase (Promega Corporation, Madison, WI, USA) following the manufacturer’s instructions.

### P. Berghei GFP Infections

GFP *P. berghei* (ANKA 2.34 strain) was maintained by serial passage in 3- to 4-week-old female BALB/c mice from frozen stocks. Mouse parasitemias were determined using light microscopy by methanol fixation of air-dried blood smears and staining with 10% Giemsa. Female mosquitoes (4–5 days old) were fed on gametocytemic mice 2–3 days after blood inoculation from infected donor mice when parasitemias were between 3–6% and 1–2 exflagellations/field. Midguts were dissected 7–8 days PI, fixed in 4% paraformaldehyde solution, rinsed in 1×PBS, and mounted in VectaShield mounting medium (Vector Laboratories Inc., Burlingame, CT, USA). Infection levels were established by counting the number of live oocysts using fluorescent microscopy; melanized parasites were detected using light microscopy. The distribution of the number of parasites that infected individual mosquitoes from the different experimental groups was compared using the nonparametric Mann-Whitney statistical test. Infection phenotypes were confirmed using 2 or 3 independent biological replicates.

### Infections with P. Falciparum 3D7 Strain

*An. gambiae* (G3) female mosquitoes were infected artificially by membrane feeding with a *P. falciparum* gametocyte culture. *P. falciparum* 3D7 strain was maintained in O^+^ human erythrocytes using RPMI 1640 medium supplemented with 25 mM HEPES, 50 mg/l hypoxanthine, 25 mM NaHCO_3_, and 10% (v/v) heat-inactivated type O^+^ human serum [26], [27]. Gametocytogenesis was induced as previously described [28]. Mature gametocyte cultures (stages IV and V) 14–16 days old were used to feed mosquitoes using warmed membrane feeders (37°C) for 30 min. Oocyst intensities in infected mosquitoes were determined by staining the dissected midguts in 0.1% mercurochrome solution and counting oocysts under light microscope. Infection phenotypes were confirmed using 2 or 3 independent biological replicates.

### Sporozoite Quantification

SGs were dissected from pools of 10–14 dsLacZ control and dsAgESP-silenced mosquitoes and homogenized in a total volume of 100 µl of PBS using a mini glass tissue homogenizer (Kontes Glass Co., Vineland, NJ, USA). Sporozoites were counted by light microscopy using a hemocytometer with the method described by Pinto et al. [17]. Infection phenotypes were confirmed using 2 or 3 independent biological replicates.

### RNA Extraction and Real-time PCR

Midguts or SGs from pools of 10–12 mosquitoes were dissected and placed in RNAlater. Total RNA was extracted using the RNeasy kit (Qiagen), and cDNA was synthesized using the QuantiTect reverse transcription kit (Qiagen) that includes a genomic DNA removal step. Gene expression was assessed by SYBR green quantitative real-time PCR (qPCR) (DyNAmo HS; New England Biolabs, Beverly, MA, USA) in a Chromo4 system (Bio-Rad, Hercules, CA, USA). PCR involved an initial denaturation at 95°C for 15 min, 44 cycles of 10 sec at 94°C, 20 sec at 56°C, and 30 sec at 72°C. Fluorescence readings were taken at 72°C after each cycle. A final extension at 72°C for 5 min was completed before deriving a melting curve (70–95°C) to determine the quality of the amplicon. All qPCR measurements were made in duplicate. Relative quantitation results were normalized with *An. gambiae* ribosomal protein S7 as internal standard and analyzed by the 2^–ΔΔCt^ method [29]. The complete set of genes screened with corresponding qPCR primers is listed in Table S1. All expression analysis was confirmed in 2–3 independent biological replicates.

### Midgut and SG Immunostaining and Confocal Microscopic Analysis

Midguts were dissected from adult female mosquitoes fed on sugar or 28 h after feeding them on healthy (C  =  control) or *Plasmodium*-infected (I  =  infected) mice. The midgut contents were removed, and the ME was fixed at room temperature in 4% paraformaldehyde as previously described [5]. Midguts were permeabilized and blocked by incubating with PBT (PBS with 1% BSA and 0.1% Triton X-100) at room temperature. Samples were incubated overnight at 4°C with anti-AgESP rabbit polyclonal serum (1∶300) (described above) or anti-PbS21 mouse mAb (1∶300). Midguts were washed three times with PBT, incubated for 3 h at room temperature with the secondary antibody (1∶300) (Alexa 488-conjugated or Alexa 555-conjugated anti-mouse or anti-rabbit antibodies; Molecular Probes; Invitrogen). After two washes in PBT and one in PB (PBS with 1% BSA), midguts were incubated with Alexa-488- or Alexa-555-labeled phalloidin (Molecular Probes) (1∶40 dilution in PB) for 20 min. SGs were dissected from adult females fed on sugar or fed on mouse infected with GFP-*P. berghei* at 18 days PI. SGs were stained using the same protocol and antibodies described above (for midguts). Tissues were mounted in VectaShield containing DAPI (Vector Laboratories, Inc.). Microscopy was performed using a Leica SP2 confocal microscope.

### Experimental Description and Statistical Analysis

Each experiment in this study was performed as three or more independent replicates. The unpaired two-tailed *t*-test was used to compare mRNA expression profiles in different experimental groups. The median intensities of oocyst and ookinete distributions were compared using the Mann-Whitney test.

## Supporting Information

Figure S1 **Effect of** ***Plasmodium falciparum*** **salivary gland (SG ) infection on AgESP expression.** AgESP mRNA levels in SGs of mosquitoes fed on a healthy (C, control; grey bar) or on a *Plasmodium berghei-*infected mouse (I, infected; red bar) 18 days post feeding. The unpaired two-tailed *t*-test was used to compare the different experimental groups (**, *P *< 0.01). All expression analysis was confirmed in 2–3 independent biological replicates.
(TIF)

Figure S2 **Effect of AgESP silencing on gelsolin expression in salivary glands (SGs).** Effect of LacZ or AgESP dsRNA injection on AgESP (blue bars) and gelsolin (red bars) mRNA levels in SGs. *Plasmodium berghei-*infected mosquitoes were injected with dsRNA 14 days post feeding, and the SGs were collected 4 days later. The unpaired two-tailed *t*-test was used to compare the different experimental groups (***, *P* < 0.001). All expression analysis was confirmed in 2–3 independent biological replicates.
(TIF)
