# Supplementary figures and images for: An Epithelial Serine Protease, AgESP, Is Required for Plasmodium Invasion in the Mosquito Anopheles gambiae
Source: PLoS One. 2012 Apr 11;7(4):e35210. doi: 10.1371/journal.pone.0035210 (PMC3324419; doi:10.1371/journal.pone.0035210)

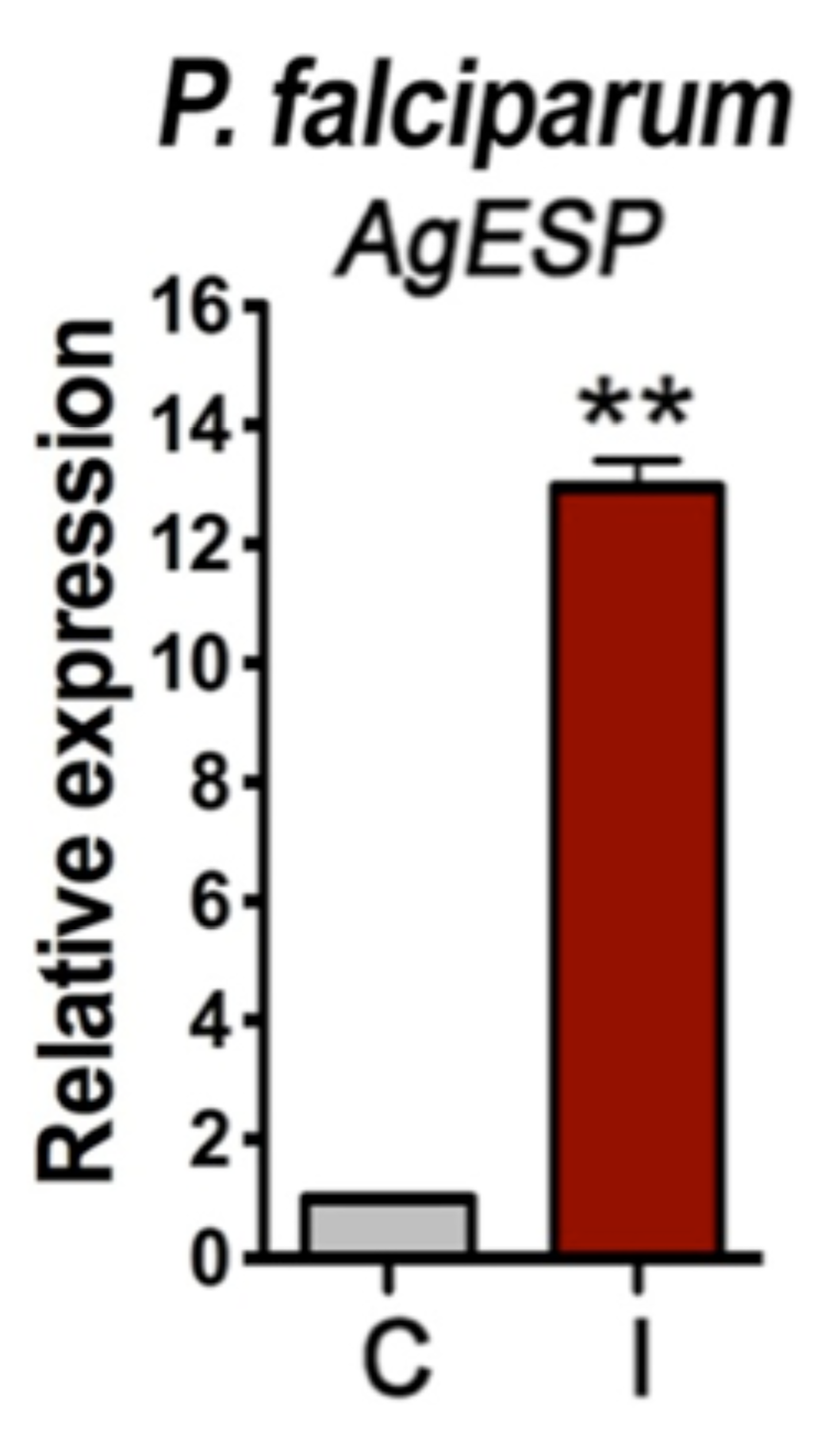

Supplement: Figure S1 — Effect of Plasmodium falciparum salivary gland (SG ) infection on AgESP expression. AgESP mRNA levels in SGs of mosquitoes fed on a healthy (C, control; grey bar) or on a Plasmodium berghei-infected mouse (I, infected; red bar) 18 days post feeding. The unpaired two-tailed t-test was used to compare the different experimental groups (**, P < 0.01). All expression analysis was confirmed in 2–3 independent biological replicates. (TIF) [file pone.0035210.s001.tif]

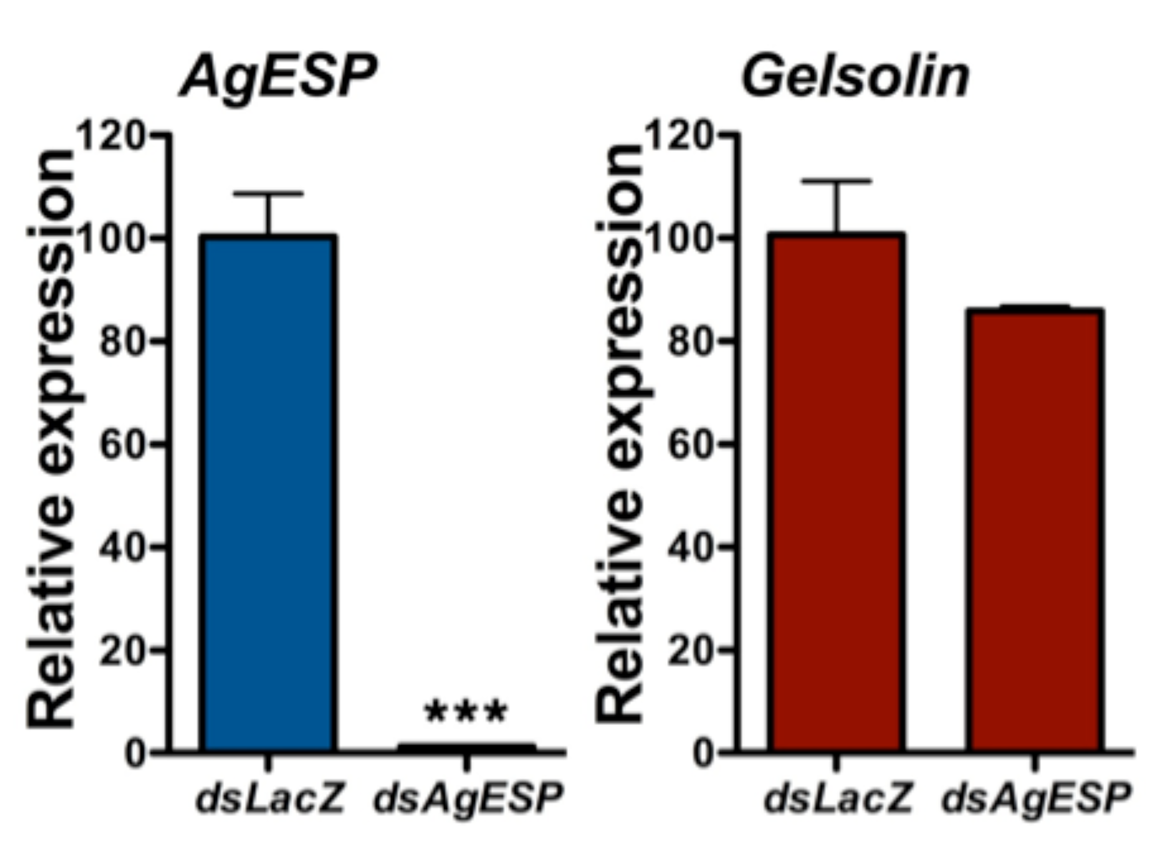

Supplement: Figure S2 — Effect of AgESP silencing on gelsolin expression in salivary glands (SGs). Effect of LacZ or AgESP dsRNA injection on AgESP (blue bars) and gelsolin (red bars) mRNA levels in SGs. Plasmodium berghei-infected mosquitoes were injected with dsRNA 14 days post feeding, and the SGs were collected 4 days later. The unpaired two-tailed t-test was used to compare the different experimental groups (***, P < 0.001). All expression analysis was confirmed in 2–3 independent biological replicates. (TIF) [file pone.0035210.s002.tif]
